# Adversarial CAM Guidance for Chest X-Ray Classification: Reducing Framing Sensitivity with Mask Supervision

**DOI:** 10.3390/biomimetics11060409

**Published:** 2026-06-10

**Authors:** Ganbayar Batchuluun, Sung Jae Lee, Su Jin Im, Kang Ryoung Park

**Affiliations:** Division of Electronics and Electrical Engineering, Dongguk University, 30 Pildong-ro 1gil, Jung-gu, Seoul 04620, Republic of Korea

**Keywords:** bio-inspired learning, framing sensitivity, chest X-ray classification, class activation mapping, discriminator-guided attention

## Abstract

The deep classifiers used for chest X-ray diagnosis can be sensitive to the visual frame around the evidence, producing correct labels for the wrong reasons by relying on the background, borders, text marks, or acquisition artifacts. This framing sensitivity reduces their trustworthiness, and they can fail under a distribution shift. Inspired by biological vision, especially figure–ground segregation and selective attention, we propose a bio-inspired adversarial attention alignment training process that encourages evidence-centered decisions without changing the classifier structure. A classifier is first trained with image-level labels. Class activation mapping (CAM) is then used to produce a differentiable heatmap that indicates where the model attends. We treat this heatmap as a generated localization map and train a discriminator to distinguish the generated heatmaps from ground truth masks. The classifier is updated using a joint objective that preserves the classification performance while pushing its CAM heatmap toward a mask-like structure, reducing the reliance on background cues. We also introduce evaluation measures for the test phase, including augmentation inconsistency (prediction flip rate under angle-based augmentations) and framing sensitivity (CAM energy outside the mask). The experiments show improved lung-focused attention and robustness, while requiring masks only during training and no additional inputs at inference.

## 1. Introduction

Deep neural networks have achieved strong performance in chest X-ray classification, including multiclass recognition of COVID, viral pneumonia, lung opacity, and normal findings. However, high accuracy does not guarantee trustworthy decision making. In medical imaging, models can exploit shortcut cues that correlate with labels but are not clinically meaningful, such as scanner-specific patterns, borders, radiographic markers, text annotations, compression artifacts, or background structures introduced by acquisition and preprocessing. When the decision depends too much on these surrounding cues rather than the true pathological evidence, the model becomes vulnerable to changes in context. This phenomenon can be described as framing sensitivity, the tendency of a model’s prediction to change when the visual frame around the evidence changes, even if the evidence itself is preserved.

Framing sensitivity matters because chest X-ray deployment conditions vary widely across hospitals, devices, and protocols. A model that is not evidence centered can fail under a distribution shift, produce unstable predictions, and reduce clinical confidence. Standard training with image-level labels provides no explicit incentive for a network to focus on a lesion or organ region. As a result, two models with similar classification accuracy can differ significantly in what they attend to. This motivates training methods that explicitly constrain attention, not merely the final label.

Bio-inspired vision offers a useful perspective. Biological perception is not a uniform processing of the entire image. Instead, it emphasizes figure–ground segregation, selective attention, and context suppression. Humans can recognize objects while discounting irrelevant background, and can maintain stable judgments under changes in framing. Translating this idea into learning objectives suggests that a classifier could be encouraged to align its internal evidence with the region that truly supports the decision, and to avoid relying on surrounding context.

A common way to inspect where a classifier attends to is class activation mapping (CAM) [1], which produces a heatmap indicating the influential spatial locations for a given class. CAM is widely used for interpretability, but it is often applied only after training as a diagnostic tool. In this work, we use CAM during training as a differentiable signal that can be shaped. Specifically, we treat the CAM heatmap as a generated localization map produced by the classifier, and we introduce an adversarial mechanism that pushes this generated map toward the structure of a ground truth mask. This creates a bio-inspired training signal that encourages figure–ground separation in the classifier’s internal reasoning.

Our proposed framework has two components. The first is a conventional classifier trained with cross-entropy using image-level labels. The second is a discriminator that receives either a CAM heatmap or a ground truth mask corresponding to the same input image. The discriminator learns to distinguish the real masks from generated heatmaps. The classifier is then updated again with an adversarial objective that encourages its CAM heatmaps to appear mask like to the discriminator while still maintaining correct classification. This training can be viewed as a generator–discriminator relationship, where the classifier acts as both a classifier and a generator of attention maps. Importantly, the method does not require masks at the inference time, only during training.

To evaluate the effect on framing sensitivity, we propose a testing protocol that perturbs the background frame while preserving the masked evidence, and we measure the prediction stability across these controlled variations.

The contributions of this paper are as follows. First, we introduce a bio-inspired adversarial attention alignment method that uses CAM as a trainable output and mask supervision as a realism constraint through a discriminator. Second, we define an evaluation approach for framing sensitivity in chest X-ray classification based on controlled frame perturbations and prediction stability metrics. Third, we demonstrate that the proposed training improves attention localization and reduces framing sensitivity while preserving classification performance, offering a practical pathway toward more robust and interpretable medical imaging models.

Our Main Contributions:

• Bio-inspired framing sensitivity definition for chest X-rays:
  We define framing sensitivity as the prediction dependence on the surrounding context when the diagnostic evidence is unchanged, motivated by bio-inspired figure–ground segregation and selective attention.

• Discriminator-guided CAM alignment as a training process:
  We introduce a training strategy where the CAM heatmaps produced by the classifier are treated as generated evidence maps, and a discriminator learns to distinguish these heatmaps from ground truth masks to provide evidence-centered guidance.

• Joint optimization for accuracy and evidence focus:
  We optimize the classifier with a combined objective that preserves the classification performance while encouraging a mask-like CAM structure, improving the interpretability without requiring masks at inference.

• Evaluation metrics for robustness and framing effects:
  We propose test-phase measures, including augmentation inconsistency (flip rate under angle-based augmentations) and framing sensitivity based on CAM–mask attention outside the evidence region.

• Model agnostic and practical applicability:
  The proposed training process can be applied to standard chest X-ray backbones and datasets, requiring masks only during training and leaving the original network structure unchanged.

• No inference overhead and practical deployment:
  The proposed training process does not modify the structure of existing models, so the inference-time processing speed, parameter count, and memory requirements remain unchanged. The method improves attention concentration and test performance, while introducing additional computation only during training due to the discriminator-guided updates.

• Reproducibility:
  Our implementation procedure and trained models are available on GitHub [2].

The remainder of this paper is organized as follows. Section 2 reviews the related literature. Section 3 describes the proposed method. Section 4 presents the experimental results and analysis. Section 5 discusses the findings, and Section 6 concludes the study.

## 2. Related Work

### 2.1. Chest X-Ray Classification and Generalization

Deep learning has been widely applied to chest X-ray classification for pneumonia and related thoracic findings. During the COVID-19 period, several chest X-ray (CXR)-based models were proposed, including COVID-Net, which also emphasized interpretability and open benchmarking [3]. However, strong internal performance often does not translate into reliable external performance. Zech et al. showed that a pneumonia detection model can exhibit variable generalization across hospital systems, and that hospital identity can become a strong confounder [4]. These results have motivated robustness-oriented training and evaluation, especially when models may rely on contextual cues rather than pathology. Other recent chest X-ray classification studies have explored attention/saliency supervision and confounder-aware learning. A gaze-guided graph neural network (GazeGNN) integrated eye gaze information through a graph-based formulation to improve CXR classification performance and robustness [5]. Another study used a multi-task UNet (MT-UNet) to jointly learn saliency prediction and disease classification, explicitly encouraging spatially meaningful evidence [6]. A causal chest X-ray (causal CXR) introduced a causal perspective to reduce the influence of confounders in CXR classification [7]. In our experiments, we used these methods as SOTA comparators and show that our training process can be applied without modifying their original architectures, improving evidence-centered attention and test performance under the same evaluation protocol.

### 2.2. Shortcut Learning and Spurious Correlations

The broader concept of shortcut learning explains why deep networks can appear accurate while exploiting non-causal cues. Geirhos et al. described shortcut learning as a pervasive failure mode in deep learning, where models adopt decision rules that work on standard benchmarks but do not transfer under distribution shifts [8]. In chest radiography, DeGrave et al. demonstrated that COVID-19 detectors can rely on confounding signals, such as text markers and border artifacts, rather than medically relevant regions [9]. Framing sensitivity in our work is closely related to these findings, but focuses specifically on how predictions and attention shift with changes in the surrounding frame.

### 2.3. Interpretability and Class Activation Mapping

CAM provides spatial explanations by linking class scores to convolutional feature maps. Zhou et al. introduced CAM using global average pooling to produce discriminative localization maps with only image-level supervision [1]. Grad-CAM extended this idea to a broader set of architectures using gradient-based weighting, and became a widely used tool for auditing model attention in medical imaging [10]. In many studies, CAM-style maps have been used post hoc for visualization. In contrast, our approach uses CAM as a trainable signal, pushing the model toward evidence-centered attention rather than treating explanations as a passive diagnostic output.

### 2.4. Adversarial Learning for Alignment of Generated Outputs

Generative adversarial nets (GANs) formalize learning through a discriminator that distinguishes real from generated samples, while the generator learns to fool the discriminator [11]. This adversarial principle has been adapted beyond image synthesis, including in settings where the generated output is a structured representation rather than a natural image. Our framework follows this direction by treating CAM heatmaps as generated localization maps and using a discriminator trained against ground truth masks to encourage a mask-like structure. This aligns with the bio-inspired goal of figure–ground segregation, where attention is guided toward the target region and away from contextual frame cues. We note that mask generation itself is commonly studied with segmentation architectures such as U-Net [12], which motivates the use of mask priors for attention guidance.

## 3. Proposed Methodology

### 3.1. Problem Setting and Goal

We consider a multiclass chest X-ray classification task with training samples (X,y,M), where $X\in R^{H\times W}$ is a chest X-ray image, y∈{1,…,K} is the image-level class label, and $M\in \{0,1{\}}^{H\times W}$ is a binary mask that marks the target evidence region. Our goal is to learn a classifier whose decision is driven by the evidence region rather than the surrounding frame, reducing the framing sensitivity. The method is bio-inspired in the sense that it encourages figure–ground segregation, pushing attention toward the figure (masked evidence) and suppressing dependence on the background frame.

**Figure 1:**
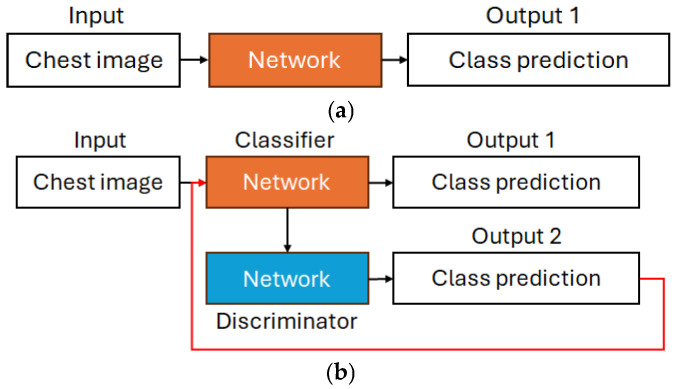
Flowchart of the proposed method. (**a**) The overarching concept and (**b**) the training phase.

Figure 1a illustrates the overarching concept, showing that the original classifier network remains structurally unchanged during inference but benefits from the updates derived from our method. Figure 1b delves into the training phase, where we introduce an external discriminator network inspired by GANs. In this setup, the discriminator guides the classifier to refine its focus toward the relevant object regions in the input image by leveraging the loss functions that align the classifier’s attention with meaningful areas. In short, the classifier is trained not only to classify but also to learn where to attend, resulting in more accurate and explainable outcomes.

The proposed framework couples a conventional classifier with a discriminator through a CAM-based attention signal. The classifier is trained in the standard supervised way to predict *y* from *X*. From the classifier, we compute a CAM heatmap *H* for a target class. This heatmap is treated as a generated localization map that represents where the classifier “looks” to make its decision. A discriminator is trained to distinguish the generated CAM maps from the ground truth masks. The classifier is then updated again with an adversarial objective so that its CAM maps become indistinguishable from real masks, while preserving the classification performance. This adversarial alignment discourages spurious reliance on the background and directly reduces the framing sensitivity by forcing the classifier’s attention to stay within the object region.

The method consists of three interacting modules.

1. Classifier $C_{\theta }$
  A backbone network extracts the feature maps *F*, followed by global pooling and a linear classifier producing the class probabilities $p=C_{\theta }(X)$.
2. CAM generator induced by the classifier
  Given *X* and a target class, the classifier produces a CAM heatmap H=CAM(X). No separate generator network is introduced; the classifier plays a dual role, both classifier and heatmap generator.
3. Discriminator $D_{\phi }$
  The discriminator receives a single channel map and outputs a probability indicating whether the map is a real mask or a generated CAM map. It is trained using mask maps as real samples and CAM maps as fake samples.

Training alternates between two updates within each iteration.

• Discriminator update:
  The discriminator learns to classify the ground truth masks as real and the CAM maps as fake.
• Classifier update:
  The classifier is optimized with a combined loss consisting of standard classification loss and an adversarial loss that encourages the discriminator to classify the CAM maps as real.

**Figure 2:**
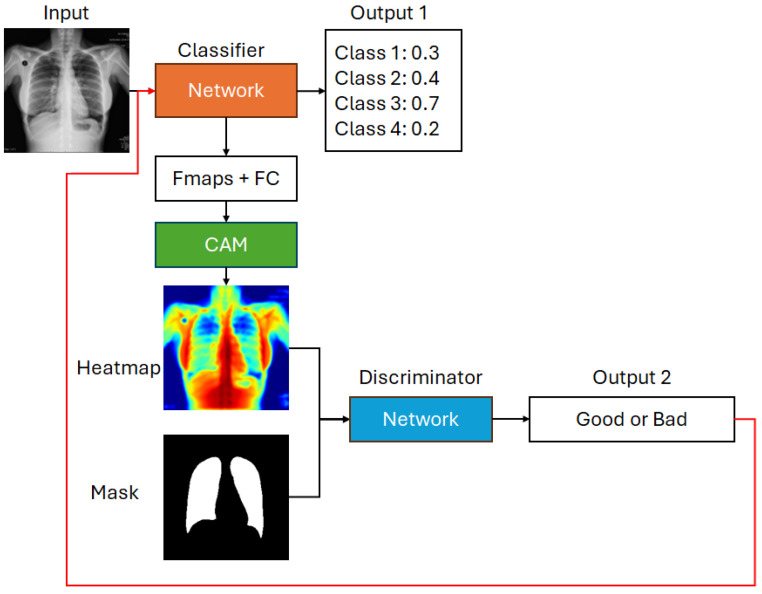
Flowchart of the proposed method during the training phase.

The chest X-ray image goes into the classifier, producing a class prediction and feature maps. These feature maps generate the CAM, highlighting what the model focused on. If the heatmap does not focus on the lung region (but instead on irrelevant areas), the discriminator comparing the CAM with the true mask helps refine the classifier. When the classifier fails to focus correctly, loss functions tied to the discriminator’s judgment update the classifier, ensuring it learns to focus on the lungs. Essentially, the process uses the CAM as a generator, and the discriminator with masks acts as a corrective guide, iteratively improving the focus on medically relevant areas. In Figure 2, “Good or Bad” corresponds to the conventional “Real or Fake” or “1 or 0” decision, and the red arrow indicates the process of refining or updating the classifier network based on the discriminator’s output.

The discriminator $D_{\phi }$ is implemented as a lightweight convolutional network that receives a single-channel map (either a ground truth mask *M* or a CAM heatmap *H*) and outputs a scalar probability indicating “real mask” versus “generated heatmap”. It consists of a small stack of 3 × 3 or 4 × 4 convolutional layers with a stride of 2, which progressively reduces the spatial resolution. The model is composed of only four convolutional layers, each followed by batch normalization and LeakyReLU, along with a global pooling layer and a final fully connected layer with a sigmoid unit for binary classification. We chose this simple CNN design because the discriminator’s role is not to model complex image content, but to learn the spatial statistics that distinguish mask-like attention maps from diffuse or background-biased heatmaps. A compact discriminator reduces the computational overhead and helps stabilize adversarial training while providing sufficient capacity to guide the classifier toward evidence-centered CAM localization.

This discriminator architecture is not a contribution of this paper; it is used as a practical example to realize the proposed training process. In principle, $D_{\phi }$ can be replaced by other discriminator designs in future implementations. Our main contribution is the training strategy, showing that adding discriminator-guided CAM alignment can improve evidence-centered attention and classification performance of existing X-ray models without modifying their original network structures. Because the proposed training strategy improves attention and classification performance without changing the structure of the existing models, it does not increase the processing time during the test phase or the model size in terms of memory requirements.

The proposed method is explained using the following equations:

(1) Classifier (backbone + GAP + linear head)

Backbone feature map:

(1) $$F_{i}(u,v,c)=f_{\theta_{b}}(X_{i})\in R^{h\times w\times d}$$

where

$X_{i}$: Input X-ray image for sample *i*.
$f_{\theta_{b}}(\cdot )$: Backbone network (convolutional feature extractor) with parameters $\theta_{b}$.
$F_{i}(u,v,c)$: Feature value at spatial location (u,v) and channel *c*.
*h*, *w*: Spatial resolution of the last convolutional feature map.
*d*: Number of channels in the last convolutional feature map.

Global average pooling:

(2) $$g_{i}(c)=\frac{1}{hw}\sum_{u=1}^{h}\sum_{v=1}^{w}F_{i}(u,v,c)$$

$where\;g_{i}\in R^{d}$ is a vector summarizing each channel in $F_{i}$ by averaging over all spatial locations.

Intuition: GAP turns the “where” information into a compact descriptor of “how strongly each feature channel is present”.

Logits with linear classifier weights $W\in R^{d\times K}$:

(3) $$z_{i}=g_{i}W\in R^{K};\;z_{i}(k)=\sum_{c=1}^{d}g_{i}(c)\;W(c,k)$$

where $W\in R^{K}$ is the weight matrix of the final fully connected (FC) layer. $z_{i}(k)$ is the logit for class *k*. Logits are the raw, unnormalized class scores produced before softmax.

Softmax probabilities:

(4) $$p_{i}(k)=\frac{exp(z_{i}(k))}{\sum_{j=1}^{K}exp(z_{i}(j))}$$

where $p_{i}(k)$ is the predicted probability of class *k* for sample *i*.

Classification loss (mini-batch of size *B*):

(5) $$L_{\mathrm{cls}}=\frac{1}{B}\sum_{i=1}^{B}CE(p_{i},y_{i})=-\frac{1}{B}\sum_{i=1}^{B}logp_{i}(y_{i})$$

(2) CAM heatmap generation (target class $y_{i}$)

Take the class weight vector

(6) $$w_{y_{i}}=W(:,y_{i})\in R^{d}$$

where

$y_{i}$ is the ground truth class label (an integer).

$W(:,y_{i})$ means the column of *W* corresponding to class $y_{i}$.

$w_{y_{i}}(c)$ is the weight applied to channel *c* when forming evidence for class $y_{i}$.

Raw CAM at feature map resolution:

(7) $$A_{i}(u,v)=\sum_{c=1}^{d}F_{i}(u,v,c)\;w_{y_{i}}(c)$$

where $A_{i}(u,v)$ is a weighted sum over the channels at each spatial location.

Positive evidence only:

(8) $${\tilde{H}}_{i}(u,v)=ReLU(A_{i}(u,v))$$

Min–max normalization to [0,1]:

(9) $$H_{i}^{\mathrm{norm}}(u,v)=\frac{{\tilde{H}}_{i}(u,v)-min({\tilde{H}}_{i})}{max({\tilde{H}}_{i})-min({\tilde{H}}_{i})}$$

Resize to image resolution:

(10) $$H_{i}=Resize(H_{i}^{\mathrm{norm}},\;H,\;W)\in [0,1{]}^{H\times W}$$

We use $H_{i}$ as the generated localization map (fake sample for the discriminator).

(3) Discriminator and adversarial losses

Discriminator output:

(11) $$D_{\phi }(\cdot )\in (0,1)$$

where

$D_{\phi }$ is a discriminator network with parameters ϕ.

Input is a map (mask $M_{i}$ or heatmap $H_{i}$) and output is a scalar probability.

Interpretation: $D_{\phi }(\mathrm{map})\approx 1$ means “looks like a real mask”, and ≈0 means “looks like a generated CAM”.

Binary cross-entropy [13]:

(12) $$BCE(q,t)=-[t\;log(q)+\left(1-t\right)\;log(1-q)]$$

where

q∈(0,1) is the discriminator’s predicted probability (e.g., $q=D_{\phi }(M_{i})$).

t∈{0,1} is the target label:

     - t=1 for real masks;
     - t=0 for fake CAM maps.

Discriminator loss (real mask $M_{i}$ vs fake CAM $H_{i}$, with stop-gradient on $H_{i}$ for discriminator step):

(13) $$L_{D}=\frac{1}{B}\sum_{i=1}^{B}(BCE(D_{\phi }(M_{i}),1)+BCE(D_{\phi }(StopGrad(H_{i})),0))$$

The discriminator learns to output 1 for masks $M_{i}$ and 0 for CAM maps $H_{i}$. $StopGrad(H_{i})$ means we treat $H_{i}$ as a constant when updating the discriminator, so the gradients do not flow back into the classifier during the discriminator step.

Adversarial loss for the classifier (makes CAM be classified as real):

(14) $$L_{\mathrm{adv}}=\frac{1}{B}\sum_{i=1}^{B}BCE(D_{\phi }(H_{i}),1)$$

During the classifier step, the classifier tries to make $D_{\phi }(H_{i})$ close to 1. This pushes the CAM maps to become more mask like, meaning attention is concentrated in the evidence region rather than the background.

### 3.2. Dataset Description

**Figure 3:**
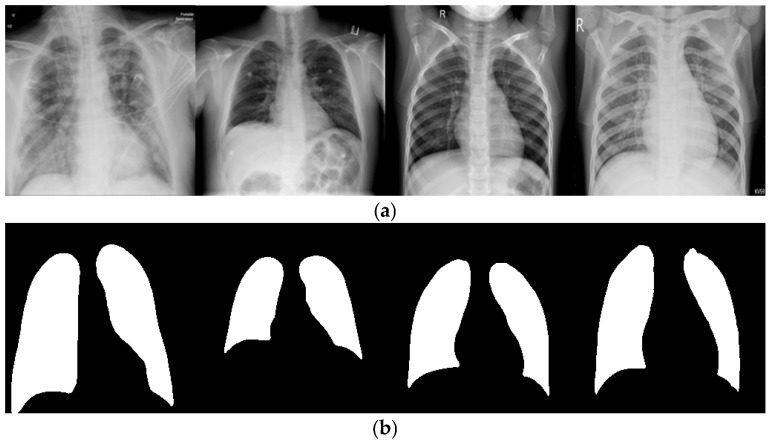
Example X-ray images from the dataset [14]. From left to right: COVID-19, lung opacity, normal, and viral pneumonia. (**a**) X-ray images and (**b**) corresponding mask images.

We used the COVID-19 Radiography Database from Kaggle [14], as shown in Figure 3, which provides chest X-ray images together with corresponding lung segmentation masks. The dataset contains four diagnostic categories: COVID-19, lung opacity (non-COVID-19 lung infection), normal, and viral pneumonia. The images are provided in the “PNG” format at a fixed resolution of “299 × 299” pixels, and the dataset includes the following class counts: 10,192 normal, 3616 COVID-19, 1345 viral pneumonia, and 6012 lung opacity, for a total of 21,165 images.

The dataset was compiled by a collaborative research team (Qatar University and Dhaka University, with additional collaborators), and the provided lung masks were generated by the dataset authors using a segmentation approach (reported as a modified U Net in prior descriptions of the database). In our project, these masks define the evidence region used to train the discriminator and to guide the classifier’s CAM maps toward object-focused attention. The masks are used only during training, and inference requires only the chest X-ray image.

For implementation, we organized the data into training, validation, and test splits, with four class folders, each containing an “images” directory for the X-rays and a “masks” directory for the corresponding ground truth masks. Each image was paired with its mask by a filename within the same class directory.

In our experiments, we balanced the class distribution to reduce the effect of class imbalance. Each class was adjusted to contain 5000 images. For classes containing more than 5000 images, extra images were removed. For classes containing fewer than 5000 images, data augmentation was applied, including horizontal flipping, left and right in-plane rotation by 20 degrees, brightness adjustment, and blurring. Therefore, the final class distribution used in the experiments was homogeneous and within an acceptable interval for classification evaluation.

After class balancing, each class contained 5000 images, resulting in a total of 20,000 images across the four classes. To reduce split-dependent bias, a two-fold validation strategy was used. The balanced dataset was divided into two equal subsets of 10,000 images each.

In the first fold, the first 10,000 images were used for training, while the second 10,000 images were used as the held-out evaluation portion. From this held-out portion, 1000 images were separated and used as the validation set during training, while the remaining 9000 images were used as the final test set. In the second fold, the procedure was reversed: the second 10,000 images were used for training, and the first 10,000 images were used as the held-out evaluation portion. Again, 1000 images were used for validation and 9000 images were used for testing.

The validation set was used only to monitor the training behavior and did not directly update the model parameters. The test set was kept separate from the training process and was used for final performance evaluation. This reverse two-fold procedure allowed both halves of the balanced dataset to be used once for training and once for held-out evaluation.

## 4. Experimental Results

### 4.1. Training Setup

This subsection describes the software environment, hardware specifications, training hyperparameters, and training setup used in this study. Table 1 presents the software environment and computer specifications, while Table 2 summarizes the hyperparameters and training configuration of the proposed method.

As shown in Table 2, the symbol “#” is used in this manuscript to indicate “the number of.” The software versions used in this study are not necessarily the newest releases. Instead, they were selected based on their compatibility with our GPU device, deep learning framework, and required implementation functions. Some functions and dependencies used in the proposed method were not fully compatible with newer versions of Python and TensorFlow/Keras. Therefore, we used stable and compatible software versions to ensure correct execution, proper GPU utilization, and reproducible experimental results.

### 4.2. Loss Functions

Classification Loss $L_{\mathrm{cls}}$: We first train the classifier in the standard supervised manner to predict the disease class from the input chest X-ray. Given the predicted class probabilities $p_{i}=C_{\theta }(X_{i})$ and the ground truth label $y_{i}$, we use cross-entropy:

(15) $$L_{\mathrm{cls}}=-\frac{1}{B}\sum_{i=1}^{B}logp_{i}(y_{i})$$

Discriminator Loss $L_{D}$: To reduce background reliance, we treat the classifier’s CAM heatmap as a generated attention map and train a discriminator to distinguish it from the ground truth mask. For each sample, the discriminator receives:

- Real: The mask $M_{i}$ (evidence region);
- Fake: The CAM heatmap $H_{i}$ produced by the classifier.

Using binary cross-entropy, the discriminator loss is defined as shown in Equation (13). We apply StopGrad to $H_{i}$ in this step so that only the discriminator is updated. This teaches the discriminator what mask-like evidence maps look like.

Adversarial Attention Loss $L_{\mathrm{adv}}$: After the discriminator update, we update the classifier so that its CAM becomes more mask like. The classifier is encouraged to fool the discriminator by making the discriminator classify the CAM heatmap as real:

(16) $$L_{\mathrm{adv}}=\frac{1}{B}\sum_{i=1}^{B}BCE(D_{\phi }(H_{i}),1)$$

Minimizing $L_{\mathrm{adv}}$ pushes the classifier’s attention to concentrate inside the object region and suppress activation in background or frame regions.

The classifier is optimized using a combined objective consisting of the classification loss and the adversarial attention loss. The classification loss preserves correct class prediction, while the adversarial attention loss encourages the classifier-generated CAM heatmap to become more consistent with the corresponding real binary lung mask. The final classifier loss is defined as

(17) $$L_{C}=L_{cls}+\lambda_{adv}L_{adv}$$

where $L_{C}$ is the total loss used to update the classifier, $L_{cls}$ is the cross-entropy classification loss, $L_{adv}$ is the adversarial attention loss obtained from discriminator feedback, and $\lambda_{adv}$ is the weighting parameter that balances classification accuracy and adversarial CAM–mask alignment.

The value of $\lambda_{adv}$ is set to 0.5 to balance classification accuracy and adversarial CAM–mask alignment. This value allows the adversarial attention loss to guide the classifier toward lung region-focused CAM heatmaps without overwhelming the main classification objective.

During training, the classifier and discriminator are updated alternately for each mini-batch. First, the input chest X-ray image is passed through the classifier to obtain the class prediction and the corresponding CAM heatmap. The generated CAM heatmap and the real binary lung mask are then used as inputs into the discriminator. The discriminator is updated to distinguish the real lung mask from the classifier-generated CAM heatmap, while the classifier parameters are kept fixed.

After the discriminator update, the classifier is updated while the discriminator parameters are kept fixed. The classifier is optimized using the combined classifier loss, which includes the classification loss and the adversarial attention loss. The classification loss encourages correct class prediction, while the adversarial attention loss encourages the generated CAM heatmap to become more consistent with the corresponding real lung mask. This alternating training process allows the discriminator to provide attention-guidance feedback to the classifier. After training is completed, the discriminator is removed, and only the trained classifier is used during the testing phase.

**Figure 4:**
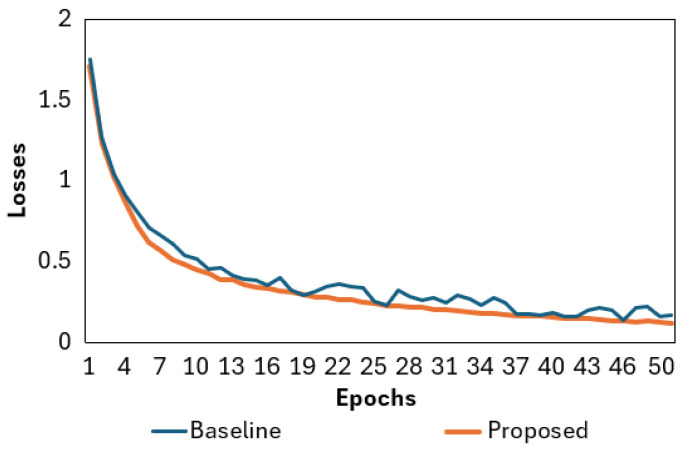
Example loss curves for comparison using DenseNet-121.

We selected DenseNet-121 to illustrate one example of loss differences across epochs during the training phase, as shown in Figure 4. Figure 4 compares the decrease in classification loss between the original DenseNet-121 training and DenseNet-121 trained using the proposed method. As shown in Figure 4, the proposed training method produces a smoother loss curve with lower loss values than the original training method.

To improve the readability of this paper, we only present the classification loss curves of one representative method, rather than showing the loss, accuracy, and validation curves of all methods used in the experiments and comparisons. Moreover, we do not compare the discriminator loss curve with the classification loss curve in the conventional manner used for comparing discriminator and generator losses, because the proposed method is a classifier method rather than a generative method.

The main objective is to retrain the classifier based on the discriminator’s feedback, not to make the generator compete with the discriminator in the conventional GAN sense. Therefore, comparing the classification loss curve of the baseline method with the classification loss curve after applying the proposed training method is the appropriate comparison.

### 4.3. Testing

#### 4.3.1. Evaluation Metrics

We evaluate the experiments using several metrics, including our newly proposed evaluation methods. To evaluate the classification performance in a conventional manner, we report the *F*1 using the true-positive rate (TPR) and positive predictive value (PPV).

(18) $$\begin{array}{c} TPR=\frac{\#TP}{\#TP+\#FN} \end{array}$$

(19) $$\begin{array}{c} PPV=\frac{\#TP}{\#TP+\#FP} \end{array}$$

(20) $$\begin{array}{c} F1=\frac{2\cdot PPV\cdot TPR}{PPV+TPR} \end{array}$$

Here, #TP, #FP, and #FN denote the numbers of true positives, false positives, and false negatives, respectively.

In addition, we evaluate the classification performance using our newly proposed evaluation methods, which measure the augmentation inconsistency and framing sensitivity. The augmentation inconsistency refers to different predictions for the same image under different angles and the framing sensitivity refers to the degree to which the decision depends on the frame surrounding the evidence. These metrics are defined using the following equations.

##### Augmentation Inconsistency

For each test image *x*, create *T* angle-based augmentations ${\left\{x^{\left(t\right)}\right\}}_{t=1}^{T}\;$(e.g., small rotations).

(21) $$AI(x)=\frac{1}{T}\sum_{t=1}^{T}1[arg\;max\;p(x)\neq arg\;max\;p(x^{\left(t\right)})]$$

Dataset score:

(22) $$AI=\frac{1}{N}\sum_{i=1}^{N}AI(x_{i})$$

where ≠ indicates “is different from”. That means:

- Compute the predicted class for the original image *x*;
- Compute the predicted class for the modified image $x^{\left(t\right)};$
- If the two predicted classes are different, the condition is true and the indicator becomes 1;
- If they are the same, it becomes 0.

##### Framing Sensitivity

The augmentation inconsistency metric is computed only from the classifier outputs p(x), measuring how the predictions change across augmentations or repeated stochastic forward passes. In contrast, the framing sensitivity metric requires spatial evidence, so we compute the CAM heatmaps *H* and compare them with the ground truth mask *M*, as in Equations (23)–(26). We first quantify the background influence using the inside and outside attention energies ($E_{\mathrm{in}},E_{\mathrm{out}}$) and the background attention ratio (BAR or $FS_{\mathrm{mask}}$). After obtaining a per-image score, we compute the dataset score by using Equations (21) and (22) across all samples.

Let H(u,v) be the CAM heatmap value at pixel location (u,v), where larger values mean the classifier is paying more attention to that pixel. Let M(u,v) be the binary mask at the same pixel, where M(u,v)=1 indicates the evidence region (lung or lesion area) and M(u,v)=0 indicates the background (frame) region.

Inside-mask energy:

(23) $$E_{\mathrm{in}}=\sum_{u,v}H(u,v)\;M(u,v)$$

This sums the CAM values only inside the mask, because multiplying by M(u,v) keeps the pixels inside the evidence region (1) and removes the pixels outside (0).

Outside-mask energy:

(24) $$E_{\mathrm{out}}=\sum_{u,v}H(u,v)\;(1-M(u,v))$$

This sums the CAM values only outside the mask, because (1−M(u,v))=1 for background pixels and 0 for mask pixels.

Background attention ratio:

(25) $$BAR=\frac{E_{\mathrm{out}}}{E_{\mathrm{in}}+E_{\mathrm{out}}+\epsilon }$$

This computes the fraction of total CAM attention that lies in the background. A lower *BAR* value means the model concentrates its attention inside the evidence region and does not rely on the frame. The small constant ϵ (e.g., $10^{-8}$) prevents division by zero.

Mask focus score:

(26) $$FS_{\mathrm{mask}}=\frac{E_{\mathrm{in}}}{E_{\mathrm{in}}+E_{\mathrm{out}}+\epsilon }=1-BAR$$

This is the complementary score, representing the fraction of CAM attention that lies inside the mask. A higher $FS_{\mathrm{mask}}$ indicates better evidence-centered focus.

Intuition: If the classifier is strongly influenced by the background or frame cues, a larger portion of the CAM heatmap will appear outside the mask, increasing $E_{\mathrm{out}}$ and therefore increasing the *BAR*. Our training aims to reduce the *BAR* and increase $FS_{\mathrm{mask}}$ by aligning the CAM heatmaps with the ground truth mask region.

#### 4.3.2. Ablation Study

In the ablation study, we evaluate the proposed training by comparing a baseline model trained with standard classification loss to the full method trained with our discriminator-guided CAM alignment. We report results for three backbones, MobileNet-v1 [21], EfficientNet-B0 [22], and DenseNet-121 [23], each trained in two settings (baseline and proposed) using the same data split and training protocol. This comparison isolates the effect of the proposed method while keeping the model structure unchanged.

For each backbone, we report *F*1 for the classification performance; the augmentation inconsistency (AI), as in Equation (22), to measure the prediction stability under angle-based augmentations in the test phase; and mask-based attention metrics that quantify the background reliance. Specifically, we compute the background attention ratio (BAR) from CAM and the ground truth mask, and its complementary mask focus score $FS_{\mathrm{mask}}=1-BAR$. A lower AI and BAR and a higher $FS_{\mathrm{mask}}$ indicate improved robustness and stronger evidence-centered attention.

Table 3 summarizes and compares the results. For each method, the “Baseline” row reports the performance under standard training, and the “Proposed” row reports the performance after applying our training process. Overall, the results show that applying the proposed training process consistently improves attention localization and stability, and can also improve classification performance, without modifying the original model structures.

**Figure 5:**
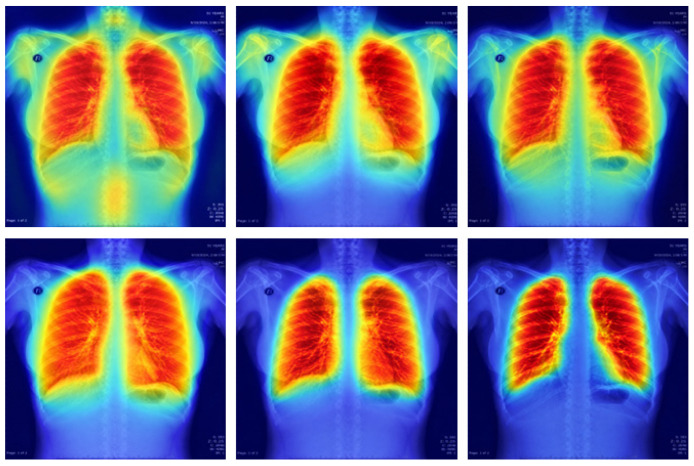
Comparison of heatmaps extracted from the methods listed in Table 3. From left to right: MobileNet-v1 [21], EfficientNet-B0 [22], and DenseNet-121 [23]. From top to bottom: baseline and proposed methods.

Figure 5 compares the heatmaps extracted from the baseline and proposed versions of the methods listed in Table 3. The visual results show that, under baseline training, several methods attend not only to the lung regions but also to surrounding chest structures and background areas. After applying the proposed training strategy, the heatmaps become more concentrated within the lung regions and show reduced activation over irrelevant areas. This indicates that the proposed method helps the compared models rely more on disease-related evidence rather than frame or background cues. These results support the quantitative findings in Table 3, where the proposed training improves the $FS_{\mathrm{mask}}$ and reduces background attention, while keeping the original model structures unchanged.

Table 4 presents the confusion matrices of DenseNet-121 as an example, comparing the baseline training and the proposed training method using the same test split. The baseline matrix shows the class-wise misclassification patterns obtained using standard classification loss, while the proposed matrix shows the results after applying our discriminator-guided CAM alignment training, without changing the network architecture. The comparison highlights that the proposed training reduces the confusion between classes and improves the overall classification performance. Table 5 compares the baseline training and the proposed method based on class-wise precision, recall, and F1-score using the DenseNet-121 backbone.

The comparison between the baseline and the proposed method represents the ablation analysis of the discriminator-guided CAM mechanism. In Table 3, Table 4, Table 5, Table 6 and Table 7, the baseline denotes training without discriminator-guided CAM heatmap alignment, while the proposed method denotes training with discriminator-guided CAM heatmap alignment. Therefore, the performance difference between these two settings directly reflects the effect of the discriminator mechanism.

Without discriminator guidance, the classifier is trained only with the classification objective, and its CAM heatmaps are not explicitly encouraged to follow the lung region. As a result, the model may still attend to irrelevant background, boundary, or frame-related regions that are not clinically meaningful. In contrast, the proposed method uses the discriminator during training to distinguish the classifier-generated CAM heatmaps from real binary lung masks. This adversarial feedback encourages the classifier to generate more mask-consistent CAM heatmaps and focus more strongly on the lung region.

The results in Table 3, Table 4, Table 5, Table 6 and Table 7 show that the proposed method improves the classification performance compared with the baseline setting. In addition, the loss curves in Figure 4 show the training behavior of the baseline and proposed models, while Figure 5 visually demonstrates that the proposed method produces CAM heatmaps that are more concentrated on the lung region. These quantitative and visual results support the necessity of the discriminator-guided CAM mechanism and show that its main effect is to improve evidence-centered attention during training without adding any inference-time complexity.

#### 4.3.3. Comparisons with X-Ray Image-Based SOTA Methods

To evaluate the generality of the proposed training process, we compared our approach with several recent X-ray image-based methods, such as GazeGNN [5], MT-UNet [6], Causal CXR [7], DenseNet201 + ViT + GAP [24], Transfer-CNN [25], and EfficientNet-CNN [26], that have been reported in the literature. For a fair comparison, we kept the original network architectures unchanged and applied our training process as an add-on optimization strategy. In other words, the compared methods were trained in their baseline form using standard classification loss, and then trained again using the same backbone and structure while adding the proposed discriminator-guided CAM alignment objective. This design allowed us to test whether the proposed bio-inspired attention guidance can improve evidence-centered focus across different architectures, rather than benefiting only a specific backbone.

Table 6 summarizes the results of this comparison. Overall, applying our training process improved attention localization and stability, and often increased the classification performance, while keeping the original model structures unchanged.

**Figure 6:**
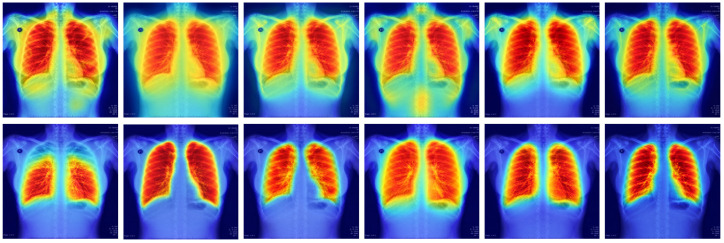
Comparison of heatmaps extracted from the methods listed in Table 6. From left to right: GazeCNN [5], MT-Unet [6], Causal CXR [7], DenseNet201 + ViT + GAP [24], Transfer-CNN [25], and EfficientNet-CNN [26]. From top to bottom: baseline and proposed methods.

Figure 6 compares the heatmaps extracted from the baseline and proposed versions of the methods listed in Table 6. This comparison also shows that the proposed method helped the compared models to focus on lung areas.

The CAM heatmaps presented in Figure 6 are used for a qualitative analysis to examine whether the proposed training encourages the classifier to focus more strongly on the lung region. However, a more concentrated CAM inside the lung area does not necessarily guarantee a correct classification result for every individual image. The classifier may attend to multiple regions, and the final decision may depend on only a subset of discriminative pixels or features. Therefore, the CAM visualization should be interpreted as an attention-localization indicator rather than direct proof of classification correctness. For this reason, the proposed method is evaluated using both quantitative classification metrics and a qualitative CAM heatmap analysis.

#### 4.3.4. Comparisons of Algorithm Complexity and Processing Time

**Figure 7:**
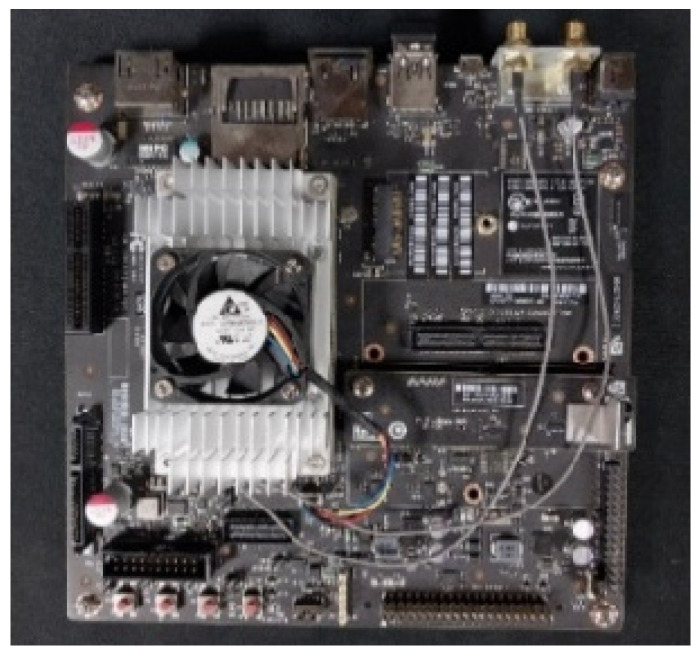
An image of the Jetson TX2 embedded system used in the experiments.

In this subsection, we analyze the computational complexity of the proposed method and the baseline methods using the desktop computer environment described in Table 1 and Jetson TX2 [27]. The NVIDIA Jetson board was selected for our experiments because it is commonly used in embedded system applications. As illustrated in Figure 7, the Jetson TX2 was equipped with an NVIDIA Pascal™ GPU containing 256 CUDA cores, 8 GB of shared CPU–GPU memory, and 59.7 GB/s memory bandwidth. Table 7 reports the processing time per image, frame per second (fps), GFLOPs, and number of parameters. As shown in Table 7, the proposed method does not increase either the processing time or the number of parameters.

#### 4.3.5. Statistical Analysis

**Figure 8:**
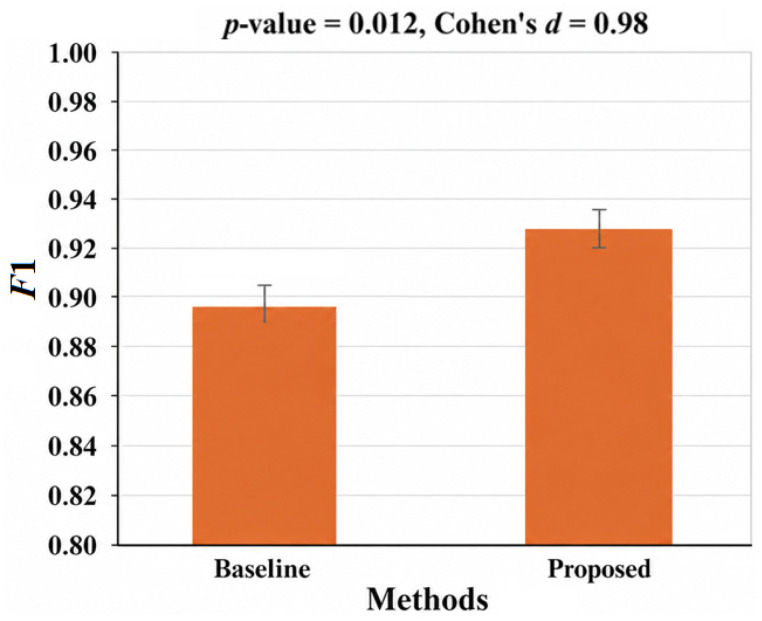
Comparison of *t*-test results for DenseNet-121 [23] based on *F*1.

In addition, we performed a statistical significance test using Student’s *t*-test [28] and quantified the effect size using Cohen’s d [29] (Figure 8). For this analysis, we compared the *F*1 obtained by the proposed training process against the baseline training under the same dataset and evaluation protocol. The statistical test produced a *p*-value of 0.012, indicating that the improvement is statistically significant at the 95% confidence level. The corresponding Cohen’s *d* of 0.98 indicates a large effect size, since values around 0.2, 0.5, and 0.8 are commonly interpreted as small, medium, and large effects, respectively. Overall, these results support that the proposed training process yields a statistically reliable improvement in the *F*1 compared with the baseline, with a strong practical impact.

## 5. Discussion

### 5.1. Information Fusion

Our training strategy can be viewed as information fusion, where multiple complementary signals are fused to guide learning beyond image-level labels. In addition to the input X-ray and class label, we fuse (1) the CAM heatmap *H* as model-derived spatial evidence, (2) the ground truth mask *M* as prior anatomical evidence, and (3) the discriminator output $D_{\phi }(\cdot )$ as a learned score that evaluates whether the heatmap is mask-like. Instead of fusing these signals at inference, we fuse them in the optimization objective, combining classification loss with discriminator-guided alignment. This fusion during training encourages the classifier to base its decision on evidence from the lungs rather than background or frame cues, improving attention localization and reducing framing sensitivity without increasing inference complexity. In our GAN-style training process, the discriminator learns to distinguish between the CAM heatmap generated by the classifier and the corresponding real binary lung mask. In contrast, the classifier acts as a generator and learns to produce CAM heatmaps that are more similar to the corresponding real lung mask. Therefore, the discriminator learns to recognize whether its input is a real mask or a classifier-generated heatmap, while the classifier learns to generate more mask-consistent attention maps. Through this adversarial process, the classifier is encouraged to focus more strongly on the lung region and reduce attention to irrelevant surrounding areas. In addition, the discriminator is used only during training to guide the classifier and is not used during the testing phase.

The main source of improvement is the proposed discriminator-guided CAM–mask alignment during training. The classifier is not changed during inference; instead, during training, its CAM heatmaps are encouraged to become more consistent with the corresponding lung masks. This adversarial guidance reduces attention to irrelevant background or frame regions and encourages the classifier to learn more evidence-centered lung-region features. Therefore, the improvement mainly comes from better attention localization and reduced dependence on surrounding non-evidence regions, rather than from adding extra parameters or changing the inference model structure.

Existing CAM-based methods are commonly used to visualize or localize discriminative regions during testing phases only, while saliency-guided learning and attention regularization usually apply direct constraints or penalties to explanation maps, gradients, or attention regions. Many attention-based methods introduce additional attention blocks, attention modules, or saliency-guided components into a model’s architecture. As a result, these methods may change the number of parameters, model size, computational complexity, and inference-time processing speed. In contrast, our proposed method guides the classifier’s attention only during the training phase through discriminator-guided CAM–mask alignment. The discriminator is removed after training, and no additional attention block or mask input is required during testing. Therefore, the final classifier keeps the original architecture, parameter count, model size, and inference-time complexity unchanged, while learning to focus more strongly on the target lung region.

The biological inspiration of the proposed method is related to figure–ground segregation and selective attention in human vision. Biological visual perception does not process all image regions with equal importance. Instead, it separates the relevant object or evidence region from the surrounding background and selectively emphasizes the meaningful visual information while suppressing irrelevant context. In the proposed method, the lung mask represents the evidence-centered figure region, while the outside-mask region represents the background or surrounding contextual information. The classifier-generated CAM heatmap represents the model’s internal attention distribution. Through discriminator-guided CAM–mask alignment, the classifier is encouraged to make its attention map more consistent with the lung region and less dependent on the surrounding frame. Therefore, the proposed mechanism can be interpreted as a computational biomimetic strategy that imitates figure–ground separation and selective attention by guiding the model to focus on anatomically relevant evidence during training.

### 5.2. Error and Correct Classification Cases

**Figure 9:**
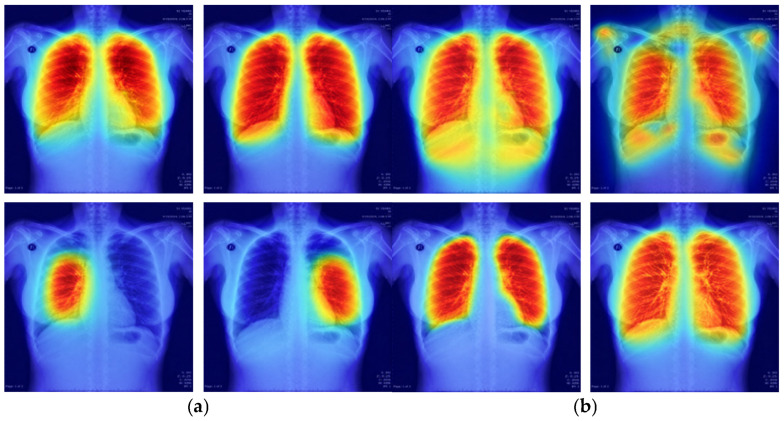
Examples of incorrectly and correctly classified images. From top to bottom: Baseline and proposed methods. (**a**) Incorrectly classified images and (**b**) correctly classified images.

Figure 9 shows correct and incorrect classification cases for the proposed method using the DenseNet-121 model. In the correctly classified cases, the proposed training reduces unnecessarily wide attention and makes the heatmap more concentrated within the lung region, which helps the model rely on more relevant evidence. In the misclassified cases, however, the attention can become too narrow, focusing on only a small part of the lung and missing other important regions. This suggests that, although the proposed method improves lung-centered attention, an overly restricted focus may still lead to classification errors.

### 5.3. Limitations and Future Work

A key limitation is that the available masks mainly indicate the lung region rather than lesion-specific pathology, so improved attention alignment does not always guarantee fully correct evidence for each class. The proposed method can also over-concentrate attention on a small lung area, which may contribute to misclassification when the discriminative cues are subtle or distributed. In addition, the discriminator-guided training requires paired masks and adds training costs, and our evaluation is based on a single public dataset, so broader external validation is still needed.

Future work will explore lesion-aware or refined evidence masks, attention constraints that avoid an overly narrow focus, and alternative discriminator designs for more stable guidance. Testing on multi-center datasets and under stronger distribution shifts will further clarify its robustness and generalization.

The proposed method relies on supervised training information. Specifically, the classifier requires image-level class labels, and the adversarial CAM guidance requires corresponding lung mask annotations during training. Therefore, the method may have limited label efficiency when sufficient labeled images or mask annotations are not available. This is an important limitation, especially in clinical settings where expert annotation can be expensive and time-consuming.

However, the rapid development of artificial intelligence-based medical image segmentation models may reduce the burden of mask annotation in future applications. Lung masks could be generated automatically or semi-automatically and then verified by experts before being used for training. In this way, the proposed CAM–mask alignment strategy could become more practical as segmentation tools continue to improve. Future work will also investigate domain adaptation strategies to improve transferability across different hospitals, imaging devices, acquisition protocols, and dataset sources while reducing the need for extensive manual annotation.

Recent domain adaptation studies in other computer vision applications have shown that domain-adaptive Faster R-CNN and transformer-based domain adaptation can improve model transferability across different image sources, supporting the potential value of this direction for reducing annotation requirements and improving robustness under a distribution shift [30,31].

The motivation for this study is not limited to the dataset used in the experiments. It is based on the broader methodological and clinical problem that chest X-ray classifiers may rely on irrelevant background or framing cues instead of evidence-centered anatomical regions. However, the experimental validation in this study was conducted on one publicly available chest X-ray dataset because the proposed discriminator-guided CAM–mask alignment process requires corresponding mask annotations together with image-level class labels. Although the dataset was suitable for evaluating the proposed training strategy, the single-dataset validation remains a limitation of this study. Future work will evaluate the proposed method on additional public and clinical chest X-ray datasets in addition to various medical image databases [32] to further examine its generalizability across different acquisition devices, hospitals, patient populations, and preprocessing protocols.

## 6. Conclusions

This paper proposes a bio-inspired training process to reduce framing sensitivity in chest X-ray classification by encouraging evidence-centered attention. Without changing the original classifier architecture, we use CAM to generate heatmaps that represent where the model focuses, and we introduce a discriminator-guided objective that aligns these heatmaps with ground truth masks during training. This guidance suppresses attention on background or frame regions and concentrates activation within the lung area.

The experiments on the COVID-19 Radiography Database show that applying the proposed training process to common backbones improves classification performance and robustness, reduces background attention (lower *BAR*), and increases mask-focused attention (higher $FS_{\mathrm{mask}}$). The qualitative heatmap comparisons further confirm that the proposed training leads to more lung-centered explanations. Overall, the results indicate that the proposed training strategy improves both the interpretability and reliability of existing chest X-ray models while keeping their structures unchanged and requiring masks only during training.

## Figures and Tables

**Table 1:** Software and hardware used in our method.

Hardware		Software	
Hardware	Specification	Library	Version
Memory	32 GB RAM	Python [15]	3.5.4
GPU	Nvidia GeForce TITAN X (12 GB)	TensorFlow [16]	1.9.0
CPU	Intel(R) Core^TM^ i7-6700 CPU@3.40 GHz (8 CPUs)	OpenCV [17]	4.3.0
		Keras API [18]	2.1.6-tf

**Table 2:** Training setup.

Parameter	Classifier	Discriminator
Loss	Categorical cross-entropy (CCE) [19]	Binary cross-entropy loss (BCE) [13]
Optimizer	Adaptive moment estimation (Adam) [20]	Adam
# Epochs	50	50
Learning rate	0.001	0.001
Batch size	4	4

**Table 3:** Comparison of baseline training and the proposed method for different backbones.

Backbone	Training	Loss Used to Update Classifier	*F*1	*AI*	*BAR*	$FS_{\mathrm{mask}}$
MobileNet-v1 [21]	Baseline	$L_{\mathrm{cls}}$	0.849	0.140	0.360	0.640
	Proposed	$L_{\mathrm{cls}}+L_{\mathrm{adv}}$	0.869	0.122	0.310	0.690
EfficientNet-B0 [22]	Baseline	$L_{\mathrm{cls}}$	0.878	0.125	0.340	0.660
	Proposed	$L_{\mathrm{cls}}+L_{\mathrm{adv}}$	0.909	0.105	0.285	0.715
DenseNet-121 [23]	Baseline	$L_{\mathrm{cls}}$	0.898	0.118	0.325	0.675
	Proposed	$L_{\mathrm{cls}}+L_{\mathrm{adv}}$	0.926	0.098	0.270	0.730

**Table 4:** Comparison of baseline training and the proposed method using confusion matrices for DenseNet-121.

	Predicted	Baseline				Proposed			
Actual		COVID-19	Lung Opacity	Normal	Viral Pneumonia	COVID-19	Lung Opacity	Normal	Viral Pneumonia
COVID-19		425	48	0	27	467	23	0	10
Lung Opacity		25	463	0	12	38	449	11	2
Normal		6	7	427	60	5	0	459	36
Viral Pneumonia		2	0	17	481	2	2	19	477

**Table 5:** Comparison of baseline training and the proposed method using class-wise precision, recall, and F1-score for DenseNet-121.

Class	Baseline			Proposed		
	PPV	TPR	F1	PPV	TPR	F1
COVID-19	0.928	0.850	0.887	0.912	0.934	0.923
Lung Opacity	0.894	0.926	0.910	0.947	0.898	0.922
Normal	0.962	0.854	0.905	0.939	0.918	0.928
Viral Pneumonia	0.829	0.962	0.891	0.909	0.954	0.931

**Table 6:** Comparison of baseline training and the proposed method for different backbones.

Backbone	Training	Loss Used to Update Classifier	*F*1	*AI*	*BAR*	$FS_{\mathrm{mask}}$
GazeCNN [5]	Baseline	$L_{\mathrm{cls}}$	0.892	0.120	0.331	0.669
	Proposed	$L_{\mathrm{cls}}+L_{\mathrm{adv}}$	0.924	0.101	0.276	0.724
MT-Unet [6]	Baseline	$L_{\mathrm{cls}}$	0.842	0.143	0.365	0.635
	Proposed	$L_{\mathrm{cls}}+L_{\mathrm{adv}}$	0.867	0.124	0.314	0.686
Causal CXR [7]	Baseline	$L_{\mathrm{cls}}$	0.881	0.127	0.346	0.654
	Proposed	$L_{\mathrm{cls}}+L_{\mathrm{adv}}$	0.910	0.108	0.292	0.708
DenseNet201 + ViT + GAP [24]	Baseline	$L_{\mathrm{cls}}$	0.872	0.119	0.318	0.682
	Proposed	$L_{\mathrm{cls}}+L_{\mathrm{adv}}$	0.895	0.101	0.264	0.736
Transfer-CNN [25]	Baseline	$L_{\mathrm{cls}}$	0.865	0.116	0.305	0.695
	Proposed	$L_{\mathrm{cls}}+L_{\mathrm{adv}}$	0.889	0.097	0.251	0.749
EfficientNet-CNN [26]	Baseline	$L_{\mathrm{cls}}$	0.858	0.111	0.298	0.702
	Proposed	$L_{\mathrm{cls}}+L_{\mathrm{adv}}$	0.884	0.093	0.243	0.757

**Table 7:** Comparison of parameters and processing time of all methods used in this study.

Backbone	Mode	Params (M)	FLOPs (G)	Inference Time per Image Unit as ms (fps)	
				Desktop	Jetson
EfficientNet-B0 [22]	Baseline	5.3	0.39	11.96 (83.61)	42.86 (23.33)
	Proposed				
DenseNet-121 [23]	Baseline	8.0	2.83	24.42 (40.95)	62.75 (15.94)
	Proposed				
MobileNet-v1 [21]	Baseline	4.2	0.56	6.96 (143.68)	35.38 (28.26)
	Proposed				
GazeCNN [5]	Baseline	10.7	1.7	19.80 (50.51)	56.50 (17.70)
	Proposed				
MT-UNet [6]	Baseline	29.0	6.2	43.00 (23.26)	128.00 (7.81)
	Proposed				
Causal CXR [7]	Baseline	44.6	7.8	52.00 (19.23)	150.00 (6.67)
	Proposed				
DenseNet201 + ViT + GAP [24]	Baseline	24.0	5.1	36.87 (27.12)	105.46 (9.48)
	Proposed				
Transfer-CNN [25]	Baseline	143.7	19.6	120.30 (8.31)	372.21 (2.69)
	Proposed				
EfficientNet-CNN [26]	Baseline	5.7	0.42	10.30 (97.04)	38.76 (25.80)
	Proposed				

## Data Availability

The original chest X-ray images and corresponding mask images used in this study are publicly available from the Kaggle dataset [14] described in the Dataset Description Section. Our implementation procedure and trained models are available on GitHub [2].
